# Microcurrent wave alleviates mouse intracranial arterial dolichoectasia development

**DOI:** 10.1038/s41598-024-58333-y

**Published:** 2024-03-29

**Authors:** Jae Hee Lee, Huy Duc Vu, Min Hee Park, Phuong Tu Huynh, Sung Won Youn, Dong Rak Kwon

**Affiliations:** 1 Department of Rehabilitation Medicine, Daegu Catholic University School of Medicine, Daegu, South Korea; 2 Department of Radiology, Kyungpook National University, Daegu, South Korea

**Keywords:** Neuroscience, Medical research, Neurology, Engineering, Nanoscience and technology

## Abstract

Intracranial arterial dolichoectasia (IADE) is associated with the interaction of hypertension and inflammation, and microcurrent can be effective in hypertension. Therefore, this study aimed to investigate the therapeutic effect of microcurrent electrical stimulation in a mouse IADE model. This study randomly categorized 20 mice into five groups: group 1-C (healthy control), group 2-D (IADE model), group 3-M + D (microcurrent administration before nephrectomy and until brain surgery), group 4-D + M (microcurrent administration for 4 weeks following brain surgery), and group 5-M (microcurrent administration for 4 weeks). Cerebral artery diameter and thickness and cerebral arterial wall extracellular matrix components were assessed. Among the five groups, group 2-D showed significantly higher cerebral arterial wall diameter (117.79 ± 17.05 µm) and proportion of collagen (42.46 ± 14.12%) and significantly lower arterial wall thickness (9.31 ± 2.26 µm) and proportion of smooth muscle cell (SMC) and elastin in the cerebral arterial wall (SMC: 38.05 ± 10.32%, elastin: 11.11 ± 6.97%). Additionally, group 4-D + M exhibited a non-significantly lower diameter (100.28 ± 25.99 µm) and higher thickness (12.82 ± 5.17 µm). Group 5-M demonstrated no evidence of toxicity in the liver and brain. The pilot study revealed that microcurrent is effective in preventing IADE development, although these beneficial effects warrant further investigation.

## Introduction

Intracranial arterial dolichoectasia (IADE) is a unique arteriopathy distinguished by its elongation, dilation, and tortuosity^1–3^. IADE demonstrated 0.1%–6.5% prevalence rates in the general population, which has increased to approximately 12% in patients with stroke^4^. IADE can frequently be asymptomatic but may present as a serious cerebrovascular disease, including cerebral infarction, cerebral aneurysm, cerebral hemorrhage, or nearby structure compressions^5–7^. Dolichoectasia-induced ischemic stroke involves two main mechanistic factors: mechanical stretching and blocking of penetrating arteries, as well as flow stagnation due to reduced flow associated with arterial dilatation. The pathogenesis of IADE has been investigated, and it can be described as a common end pathway of arterial wall damage that particularly affects the tunica media related to hypertension^6,8,9^. A previous study^1^ reported that IADE development is closely connected to hemodynamic stress-induced inflammation and extracellular matrix (ECM) remodeling within the arterial wall.

Currently, IADE is managed primarily based on the patient’s clinical presentation and the disease severity. This approach includes measures, such as blood pressure control, the use of antithrombotic agents, and in some cases, more invasive treatments, including endovascular procedures and surgery^10,11^. Antithrombotic medications are often recommended as a prophylaxis for preventing secondary ischemic strokes, but patients with dolichoectasia still experience an increased risk of recurrent ischemic strokes^3^. Surgical interventions for dolichoectatic aneurysms are challenging to approach, and they require intensive planning and caution to avoid iatrogenic rupture during the procedure^11^. Safe and non-invasive treatment options that consider the underlying pathophysiological factors to be corrected need to be explored because the optimal treatment for IADE still has no consensus.

This study considered microcurrent therapy as the potential strategy to modify the natural course of IADE because microcurrent therapy addressed hypertension and inflammation simultaneously. Microcurrent is an emerging treatment method applying extremely low-level electric currents in the microampere range to treat various medical conditions, including Alzheimer's disease^12^, tension headaches^13^, sleep disorders^14^, depression^15^, congenital muscle torticollis^16,17^, musculoskeletal problems such as back pain^18^, knee pain^19^, and shoulder pain^20^ with wound healing^21^. The exact mechanisms of microcurrent therapy remain unknown, but it is thought to be associated with increased adenosine triphosphate (ATP) production, improved amino-acid transportation, and enhanced protein synthesis, thereby reducing inflammation and promoting tissue healing^22,23^. Several previous studies have revealed the effectiveness of microcurrent in managing hypertension by mitigating oxidative stress and exhibiting anti-inflammatory effects^24,25^. Furthermore, an animal study that investigated sinusitis in mice revealed that microcurrent treatment reduced inflammatory cytokine levels when compared to untreated sinusitis mice^24^. Therefore, microcurrent therapy can be a safe and non-invasive treatment option that could target hypertension and inflammation, thereby potentially offering an alternative approach to managing IADE and improving patient outcomes. However, as far as we have searched, there have been no pre-clinical studies that evaluated the efficacy and safety of microcurrent in treating IADE and related conditions. Additionally, no previous study has applied microcurrent to IADE.

This proof-concept study aims to assess the effect of microcurrent electrical stimulation on IADE development in the induced murine model. Each sham operation, induced dolichoectasia, and several treatment groups were compared.

## Results

### Microcurrent wave alleviates IADE-related arterial morphologic changes

**Figure 1:**
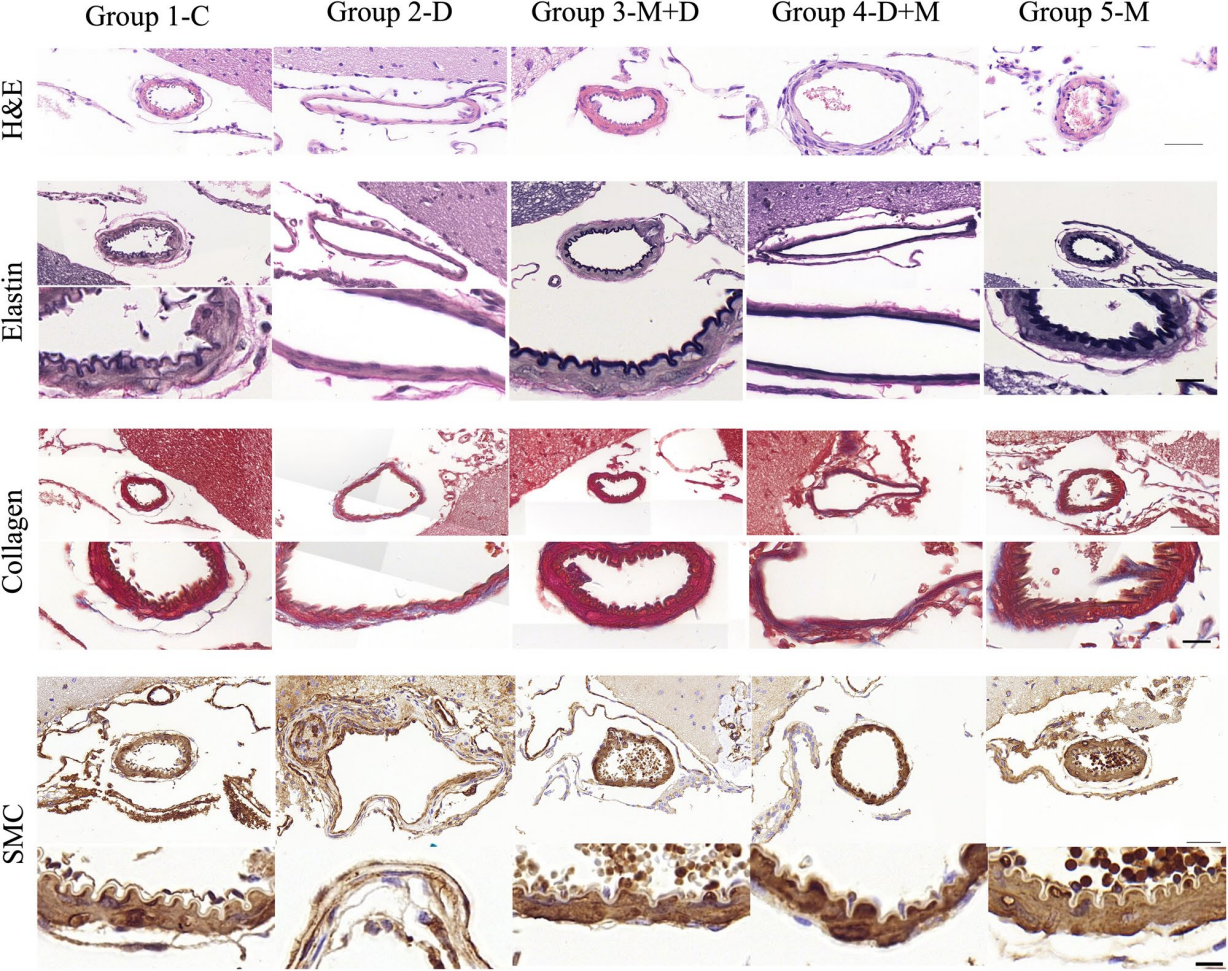
Representative Hematoxylin and Eosin (H&E) staining and immunohistochemistry staining images of each group.

**Figure 2:**
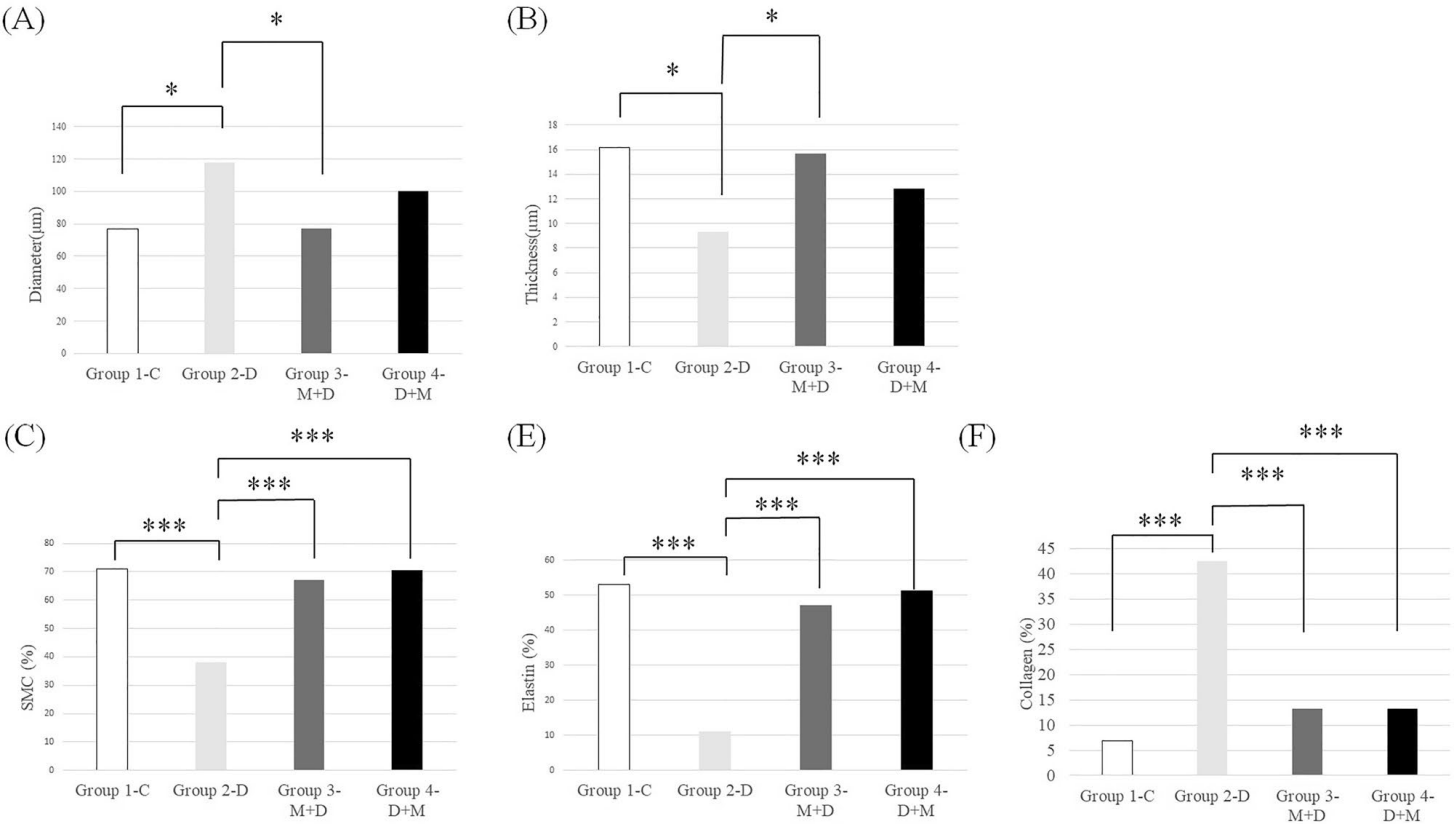
Comparison of morphologic changes of the cerebral arterial wall measured by diameter, thickness, and extracellular matrix (ECM) composition. Comparison of diameter (A) and thickness (B) of cerebral artery. Comparison of ECM composition of the cerebral arterial wall, including smooth muscle cell (SMC) (C), elastin (D), and collagen (E). *p < 0.05, one-way ANOVA and post hoc Tukey test between the groups. ***p < 0.001, one-way ANOVA and post hoc Tukey test between the groups.

Figure 1 shows the representative Hematoxylin and Eosin (H&E) images of arterial walls. The cerebral arterial lumen diameter was significantly higher in group 2-D (117.79 ± 17.05 µm) compared to group 1-C (76.64 ± 12.03 µm; *p* = 0.031) and group 3-M + D (77.29 ± 24.47 µm; *p* = 0.01). Cerebral arterial wall thickness in group 2-D (9.31 ± 2.26 µm) was significantly lower than in group 1-C (16.16 ± 1.6 µm; *p* = 0.012) and group 3-M + D (15.67 ± 2.86 µm; *p* = 0.004) (Fig. 2, Table 1). The arterial lumen diameter of group 4-D + M (100.28 ± 25.99 µm) was lower than that of group 2-D (117.79 ± 17.05 µm) (*p* = 0.641) and the arterial wall thickness of group 4-D + M (12.82 ± 5.17 µm) was higher than that of group 2-D (9.31 ± 2.26 µm) (*p* = 0.349) although with no significant difference.

**Table 1:** Comparison of the morphological changes in the cerebral artery among the five groups.

Variable	Group 1-C	Group 2-D	Group 3–M + D	Group 4–D + M	Group 5-M	*P*-value
Diameter (μm)	76.64 ± 12.03^†^	117. 79 ± 17.05^†‡^	77.29 ± 24.47^‡^	100.28 ± 25.99	67.25 ± 14.53	0.002*
Thickness (μm)	16.16 ± 1.6^†^	9.31 ± 2.26^†‡^	15.67 ± 2.86^‡^	12.82 ± 5.17	16.92 ± 2.87	0.001*
SMC (%)	70.93 ± 7.18^†^	38.05 ± 10.32^†‡♦^	67.03 ± 6.17^‡^	70.45 ± 9.35^♦^	68.21 ± 6.22	< 0.001*
Elastin (%)	53.13 ± 9.08^†^	11.12 ± 6.97^†‡♦^	47.22 ± 8.73^‡^	51.2 ± 6.82^♦^	61.26 ± 8.53	< 0.001*
Collagen (%)	6.94 ± 2.76^†^	42.46 ± 14.12^†‡♦^	13.31 ± 4.67^‡^	13.3 ± 3.84^♦^	9.4 ± 4.43	< 0.001*

### Microcurrent wave preserves smooth muscle cells (SMC) and elastin while controlling collagen production in the IADE-induced arterial wall

Group 2-D showed a significantly lower proportion of SMC and elastin in the cerebral arterial wall (SMC: 38.05 ± 10.32% and elastin: 11.11 ± 6.97%) compared to groups 1-C (SMC: 70.93 ± 7.18% and elastin: 53.13 ± 9.08%), 3-M + D (SMC: 67.03 ± 6.17% and elastin: 47.22 ± 8.73%), and 4-D + M (SMC: 70.45 ± 9.35% and elastin: 51.2 ± 6.82%) (*p* < 0.001), respectively. Group 2-D (42.46 ± 14.12%) demonstrated a significantly higher proportion of collagen in the cerebral arterial wall compared to groups 1-C (6.94 ± 2.76%), 3-M + D (13.31 ± 4.67%), and 4-D + M (13.3 ± 3.84%) (*p* < 0.001), respectively (Fig. 2, Table 1). However, the proportion of SMC, elastin, and collagen in the cerebral arterial wall demonstrated no statistically significant differences among groups 1, 3, and 4 (Fig. 2, Table 1).

### Cluster of differentiation 68 (CD68) staining and matrix metalloproteinase-8 (MMP-8) expression by immunohistochemistry

**Figure 3:**
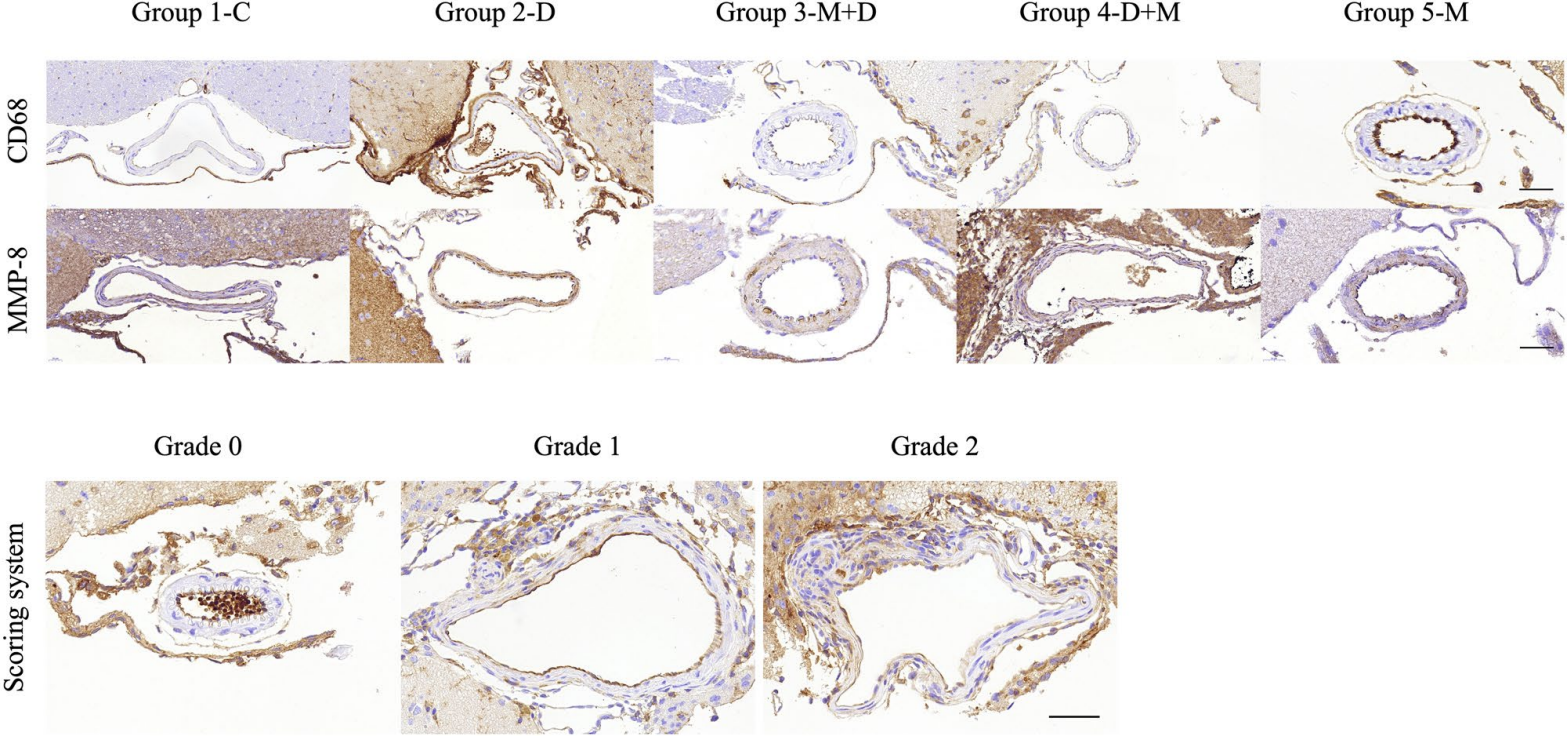
Representative immunohistochemistry staining images of each group. Immunohistochemical staining revealed distinctive expression patterns of CD68 and MMP-8 between groups 1 (grade 0/0) and 2 (grade 2/2). The expression patterns of microcurrent treatment groups were variable: group 3 (CD68/MMP-8, grade 0/2), group 4 (grade 1/1), and group 5 (grade 1/1). The staining scoring system is defined as follows: immunohistochemistry grade 0: no stain, grade 1: less than half, grade 2: more than half of arterial wall circumference.

Figure 3 shows representative immunohistochemically stained arterial walls. Immunohistochemical staining revealed distinctive high expression patterns of CD68 and MMP-8 in group 2 compared to those in group 1. Furthermore, the expression patterns of group 3 were somewhat morphologically similar to group 1, while that of group 4 was different from group 1. However, the expression patterns of CD68 and MMP-8 demonstrated no significant difference, as examined by the scoring system among the groups.

### Major organs of mouse do not show any sign of damage after microcurrent wave

Group 5-M revealed no signs of toxicity, according to histological observations in the liver and brain.

## Discussion

This study revealed that microcurrent effectively prevents IADE progression, as evidenced by the improvement in morphology, such as the diameter and thickness of the cerebral artery, and the prevention of ECM degeneration. To the best of our knowledge, this study is the first to reveal the effect of microcurrent on IADE development. Dolichoectatic arteries are characterized by a decreased arterial wall thickness and increased arterial lumen diameter, resulting in tortuous, elongated, and widened cerebral arterial configuration^5^. Previous studies that used the elastase-induced IADE mouse model revealed that arterial lumen dilatation occurs due to internal elastic lamina loss, concerning the inflammatory process and increased MMP expression. These MMPs, released by activated macrophages, digest elastin, leading to SMC apoptosis and the artery’s mechanical weakening^1,26,27^. The present study revealed that the IADE group showed dilated cerebral arteries lumen diameter and thinned cerebral arteries wall compared to the control group, which is in concordance with previous results.. Conversely, the preoperatively applied microcurrent significantly restored the morphology of the cerebral arteries compared to the IADE group. Therefore, the microcurrent can be recognized to affect abnormal cerebral artery deformation in IADE progression and homeostatically restore the abnormal arteries. These results indicate that microcurrent could be a potential therapeutic approach for managing IADE and its related cerebral artery abnormalities.

Microcurrent therapy has treated various diseases, such as musculoskeletal conditions, wounds, and pain. One of its advantages is that it provides subsensory stimulation, indicating that it is < 1 mA, which results in no discomfort for patients compared to other conventional electrical stimulations such as transcutaneous electrical nerve stimulation.^18^ Moreover, many other clinical and animal studies reported no severe adverse effects of microcurrent^16,18,19,23,28,29^. The exact mechanism of microcurrent therapy remains unclear, but it may be related to increasing the adenosine triphosphate generation, facilitating amino-acid transportation, and promoting protein synthesis to reduce inflammation and induce tissue healing.^22^ Additionally, microcurrent may regulate the disturbed homeostasis of intracellular Ca^2+^ in injured tissues, thereby reducing muscle shortening and improving maximum force production in muscles with delayed-onset muscle soreness^30^. McMakin et al.^31^ revealed that a substantial reduction of inflammatory markers, including interleukin-1 (IL-1), IL-6, tumor necrosis factor-alpha (TNF-α), and the neuropeptide substance P was observed in patients with fibromyalgia treated with microcurrent. Microcurrent therapy has been increasingly utilized for various medical conditions due to its effectiveness and non-invasiveness, along with minimal side effects and discomfort. Thus, we selected microcurrent as the treatment for IADE in our study.

Hypertension or hemodynamic stress also contributed to inducing endothelial cell dysfunction, inflammatory cell infiltrations, SMC breakdown, and ECM remodeling, ultimately leading to vessel wall degeneration and cell death^32,33^. Hence, the management of arterial hypertension is crucial in dealing with dolichoectasia. Lin et al.^34^ reported hypertension as a major contributing factor in the course of IADE formation and caused the increased incidence of ischemic stroke and cerebral hemorrhage. Cerebral infarction in IADE cases is caused by luminal thrombi blocking small branches of the arteries. Furthermore, the thin and dilated arterial wall may be broken and bulged with a developing aneurysm, which poses a risk for intracranial hemorrhage^35^. Conventional treatment for ischemic stroke involves long-term anti-platelet agents, anti-coagulation agents, or invasive interventions. However, these thrombolytic or anticoagulant therapies could be a risk factor for aneurysm rupture and intracranial hemorrhage. The advent of the new treatment concept has been anticipated because the control of hypertension becomes crucial in managing IADE which carries higher risks of ischemic stroke, cerebral aneurysm, or intracranial hemorrhage^9,34^.

Microcurrent may be a beneficial treatment option for hypertension by managing oxidative stress, which is a major factor in cardiovascular disease development. Zalba et al.^36^ indicated that various factors, such as humoral, genetic, and hemodynamic elements, activate NAD(P)H oxidase, thereby increasing the production of superoxide anion, which is associated with endothelial dysfunction and media hypertrophy. Reactive oxygen species (ROS), including superoxide anion, play a crucial role in intracellular signaling and may contribute to vascular SMC (VSMC) hypertrophy and hyperplasia. Thus, high blood pressure regulation by reducing ROS appears promising. Lee et al.^25^ revealed that microcurrent application could stabilize mitochondria, act as antioxidants, and enhance vascular tissue function, thereby potentially aiding hypertension control. A previously unpublished study revealed that microcurrent effectively lowered blood pressure by reducing liver fat metabolism caused by hyperglycemia and renin levels. The present study revealed that the preoperative application of microcurrent resulted in significantly shorter cerebral arterial diameters and thicker arterial walls compared to the IADE group. The IADE group and the group with microcurrent applied after inducing IADE demonstrated no significant difference, but the latter group exhibited a trend of lower diameter and higher thickness. Blood pressure and ROS were not assessed in this study, but these results indirectly indicate that microcurrent may affect IADE by influencing hypertension. Further research is needed to understand the exact evidence and mechanism of how microcurrent controls hypertension and its potential effects on IADE.

The results of our study revealed that SMC, elastin, and collagen components in the groups where microcurrent was applied pre- or postoperatively were maintained at similar levels to the healthy control group and significantly different compared to the IADE group. This observation is congruent with a previous study reporting that microcurrent restores the normal function of vascular tissues^25^. The arterial walls consist of ECM components, including SMC, elastin, and collagen, with elastin being a key factor in IADE formation. The single elastase injection in the cerebrospinal fluid (CSF) space reduced elastin and broke down the normal arterial wall composition. Our previous study^1^ revealed a lower proportion of elastin and higher proportions of collagen and SMC in the dolichoectactic artery. Liu et al.^37^ revealed elastin degradation and increased type 1 collagen, thereby contributing to stiffness of the arterial wall in coronary arteria ectasia. Conversely, Kapeller et al.^38^ revealed that microcurrent affects an alteration in the extracellular matrix and reduces collagen type 1 in myocardial specimens of spontaneously hypertensive rats. Additionally, microcurrent supported early wound healing by stimulating the regeneration of vessels affected by cigarette smoke, which is also a risk factor for IADE^39^. These findings indicate that the microcurrent may help maintain and normalize arterial vasculature, thereby contributing to IADE prevention and management.

Inflammation is a significant factor that influences arterial wall dilatation, and numerous studies have implicated macrophages and monocytes as important agents in inflammation and IADE formation^40–42^. Our study revealed that the IADE groups demonstrated a significantly lower elastin concentration and a higher proportion of collagen in the arterial wall. Upregulated secretion of MMP within the arterial wall seems to mediate elastic fiber degradation and reduction in elastin proportions. As elastin was progressively lost, the collagens remained highly concentrated, contributing to dolichoectasia^40^. Furthermore, Laxton et al.^43^ revealed low angiotensin I and high angiotensin II in MMP-8 abundant mice, probably indicating that MMP-8 is involved in the conversion process of angiotensin I to II. Additionally, MMP-8 functions as a generator for angiotensin II production, leading to increased blood pressure. MMP-8 is also involved in vascular inflammation through angiotensin II^44^. Therefore, the curtailment of MMP might be effective in treating IADE, considering the pathophysiology. CD68, a marker of macrophages, is also related to inflammation. Other previous studies revealed some association of CD68 with cerebral aneurysm formation and rupture^45,46^. Our previous study revealed a significant increase in CD68 in the IADE model as the inflammation occurred in the IADE formation, and treatment with melittin-loaded L-arginine-coated iron oxide nanoparticles reduced inflammation and prevented IADE formation. Our present study revealed no significant difference in the scoring system of the expression patterns of MMP-8 and CD68 between the IADE group and the microcurrent applied groups, but we observed gross morphological differences between the disease and treatment groups. The small sample size may have caused the lack of significant differences in the scoring system. Further studies with larger sample sizes are required to fully evaluate the quantitative measurements of inflammation and the mechanism of microcurrent in reducing inflammation.

The present study has several limitations. First, the sample size was relatively small, which may have affected the statistical significance of the results. Additionally, brain tissue from mice is quite small, and technical errors during tissue cutting or trimming could cause the loss of the cerebral arteries in the specimens, thereby potentially affecting the analysis. Second, we did not perform diverse frequencies and durations of microcurrent. We selected the present microcurrent type referred to in the previous study^47^, but further investigations with diverse microcurrents are needed to determine the most effective type of microcurrent therapy for IADE. Third, this study included no clinical assessment. Future studies should consider additional parameters, such as neurologic signs or symptoms for clinical application. Fourth, we did not include a group that continuously applied microcurrent from preoperation to postoperation. Such a group could provide valuable insights into the impact of all areas from prevention to microcurrent treatment. Fifth, we did not contemplate endothelial cell dysfunction and chronic inflammation markers such as hsCRP and fibrinogen. Sixth, while we noted enhancements in the tunica intima and tunica media following microcurrent therapy, our study's scope is limited by the lack of biomarker data for the tunica intima, warranting further investigation, Seventh, we relied solely on immunohistochemistry (IHC) for inflammation evaluation. Future studies might consider additional methods, such as Western blot and polymerase chain reaction, for a more quantitative evaluation of inflammation. Finally, given the safety profile and efficacy of this microcurrent therapy, Microcurrent may be a promising preemptive strategy to protect against IADE development. Further clinical feasibility studies will be required in high-risk populations for IADE development.

## Conclusion

This pilot study revealed the effectiveness of microcurrent therapy in preventing and positively affecting IADE development by improving the morphology and composition of cerebral arteries compared to the non-treatment group. These results indicate that microcurrent could be a promising and non-invasive treatment option for IADE. Further comprehensive studies are necessary to fully comprehend the mechanism underlying microcurrent therapy and its potential clinical application. Overall, this study establishes a solid groundwork for future research and clinical investigations of microcurrent therapy as a potential IADE treatment.

## Methods

### Experimental reagents

We purchased angiotensin II (A9525) and elastase (E7885) from Sigma-Aldrich (St. Louis, Missouri, USA) and Alzet osmotic pumps (200 µL, 0.5 µL/h) from Durect corporation (Cupertino, California, USA). The 10 µL model 701 syringe with a 26-G 2.0-inch point-style-3 Hamilton replacement needle was supplied by Fisher Scientific (Hampton, New Hampshire, USA). We purchased IHC antibodies from Abcam (Cambridge, UK) and Cell Signaling Technology (Danvers, Massachusetts, USA).

### Animal grouping and IADE model generation

**Figure 4:**
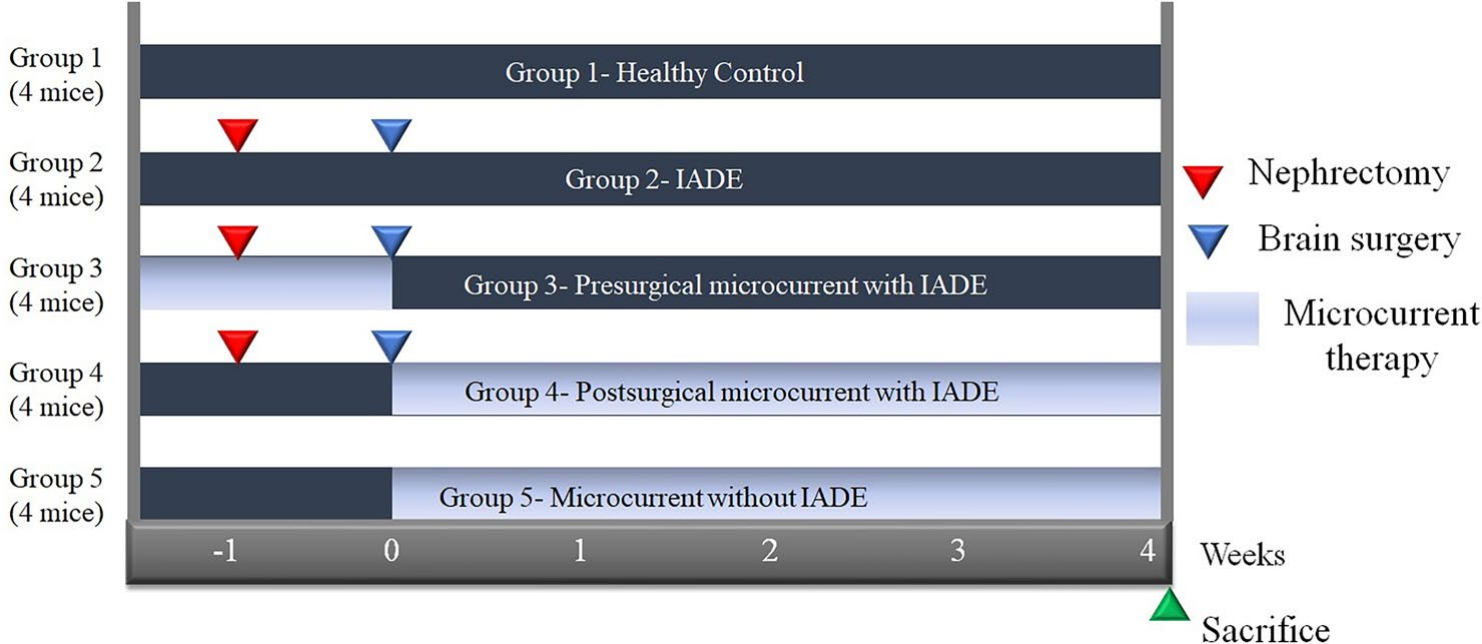
Timeline of the study. Twenty mice were randomly allocated into 5 groups: group 1 (healthy control); group 2 (the intracranial arterial dolichoectasia [IADE] model); group 3 (microcurrent therapy for a total of 2 weeks, beginning one week before nephrectomy and continuing through to brain surgery to prevent IADE); group 4 (microcurrent therapy administered for 4 weeks after the brain surgery for treatment of IADE); group 5 (microcurrent therapy administered for 4 weeks from 0th week for toxicity evaluation, when the brain surgery was performed in the other group).

This animal study was approved by the Institutional Animal Care and Use Committee (IACUC) of the Catholic University of Daegu School of Medicine (Approved number: DCIAFCR-221027-28-YR)), which is the author’s affiliation, in compliance with IACUC guidelines for the care and use of animals. In addition, this study was conducted in accordance with ARRIVE guidelines. This study randomly allocated 20 mice using computerized random numbers into five groups: group 1-C (healthy control), group 2-D (IADE model), Group 3-M + D received microcurrent therapy for a total of 2 weeks, beginning one week before nephrectomy and continuing through to brain surgery to prevent IADE, group 4-D + M (microcurrent administration for 4 weeks after brain surgery for IADE treatment), and group 5-M (microcurrent administration for 4 weeks to evaluate toxicity) (Fig. 4).

**Figure 5:**
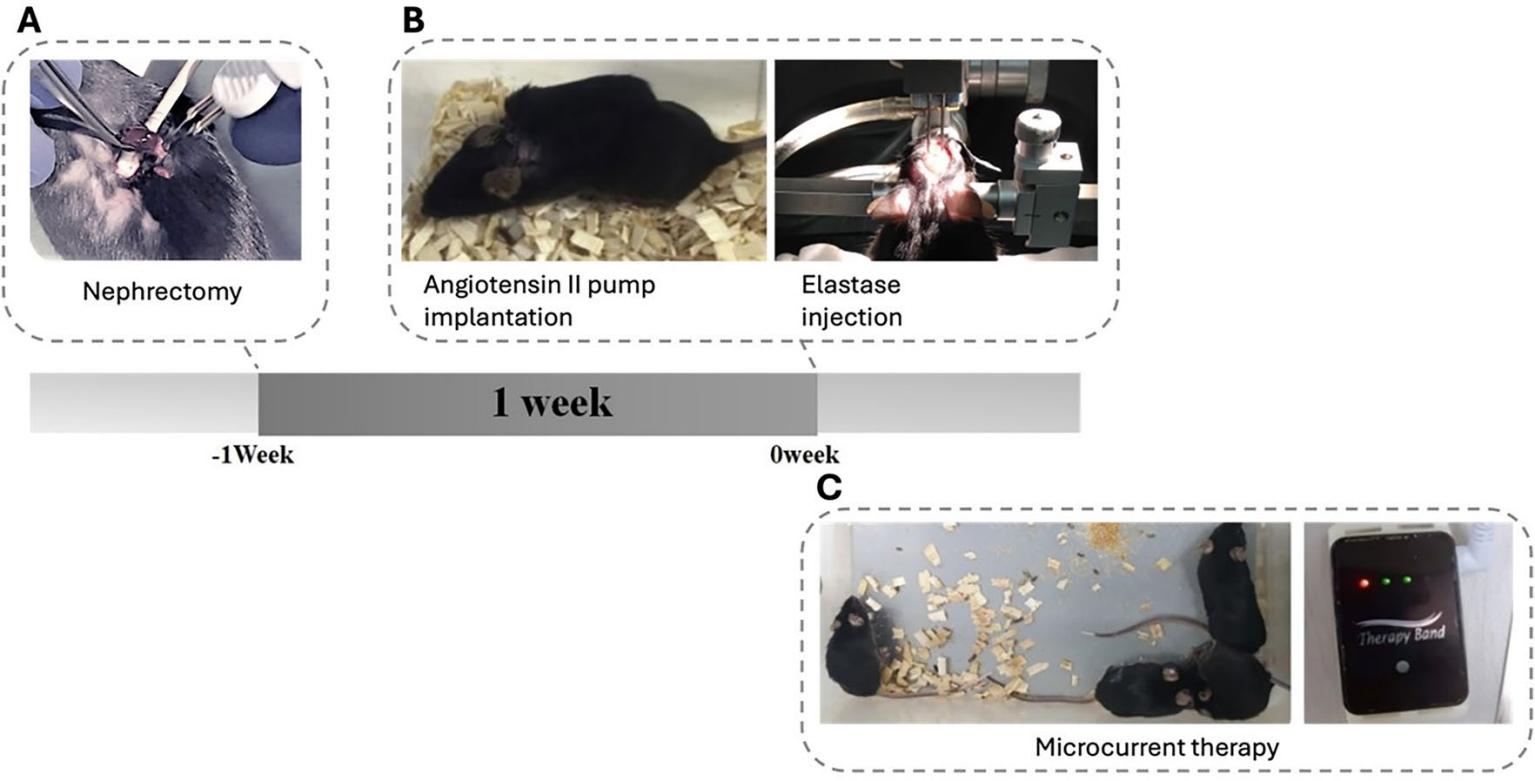
Schematic diagram of IADE model generation. A. Hypertension or hemodynamic stress was induced by unilateral nephrectomy 1 week before brain surgery. B. Elastase was injected stereotaxically into the right basal cistern and angiotensin II was continuously infused by subcutaneous osmotic pump implantation at 0 week. C. Microcurrent therapy was performed.

The 6-week-old C57/BL6 mice (Hyo-Chang Science, Korea) were used to create the IADE model. The breeding room maintained a 12-h light–dark cycle to artificially create day and night environments, with free access to normal feeding and water supply. The mice were allowed to adapt to the new environment for 1 week preoperatively. One week before the brain surgery, a unilateral nephrectomy was performed to induce hypertension or hemodynamic stress, following the procedures as outlined below. Preoperatively, the mice were anesthetized with isoflurane inhalation anesthesia. The skin on the unilateral posterolateral side of the back was shaved. An incision was made perpendicular to the spine after finding the spleen to determine the location of the kidney. The skin was then removed, and an incision was made in the abdominal wall. Light pressure was applied on either side of the incision to expose the kidney within the body wall. The left kidney was surgically removed, the abdominal wall was then sutured, and the skin wound was closed with clips (Fig. 5-A)..

The brain surgery was conducted 1 week after the nephrectomy as follows: a single dose of elastase (4 μL of 2.5 mU/μL) solution was injected stereotaxically into the ventricular zone of the mice at 0.2 μL/min. The coordinates of the ventricular zone were determined based on the mouse brain’s Atlas. The bregma was determined by the coronal suture and sagittal suture intersection. The syringe was manipulated 0.2 mm posteriorly from the bregma, 1.0 mm laterally to the right, and 2.4 mm ventrally from the skull surface. After the injection, an angiotensin II pump was subcutaneously implanted at the back of the mouse to create the IADE model (Fig. 5B).

The mice were subjected to cardiac perfusion with 4 mL of phosphate-buffered saline (PBS), 4 mL of 4% paraformaldehyde (PFA), and 4 mL of bromophenol blue dye solution dissolved in 10% (w/v) gelatine/PBS, at week 4 after microcurrent treatment. The brains were carefully isolated from the skull using forceps. The brain tissues were immersed in 4% PFA in the refrigerator (4 °C) for at least one day before conducting histopathologic analysis.

### Microcurrent therapy

**Figure 6:**
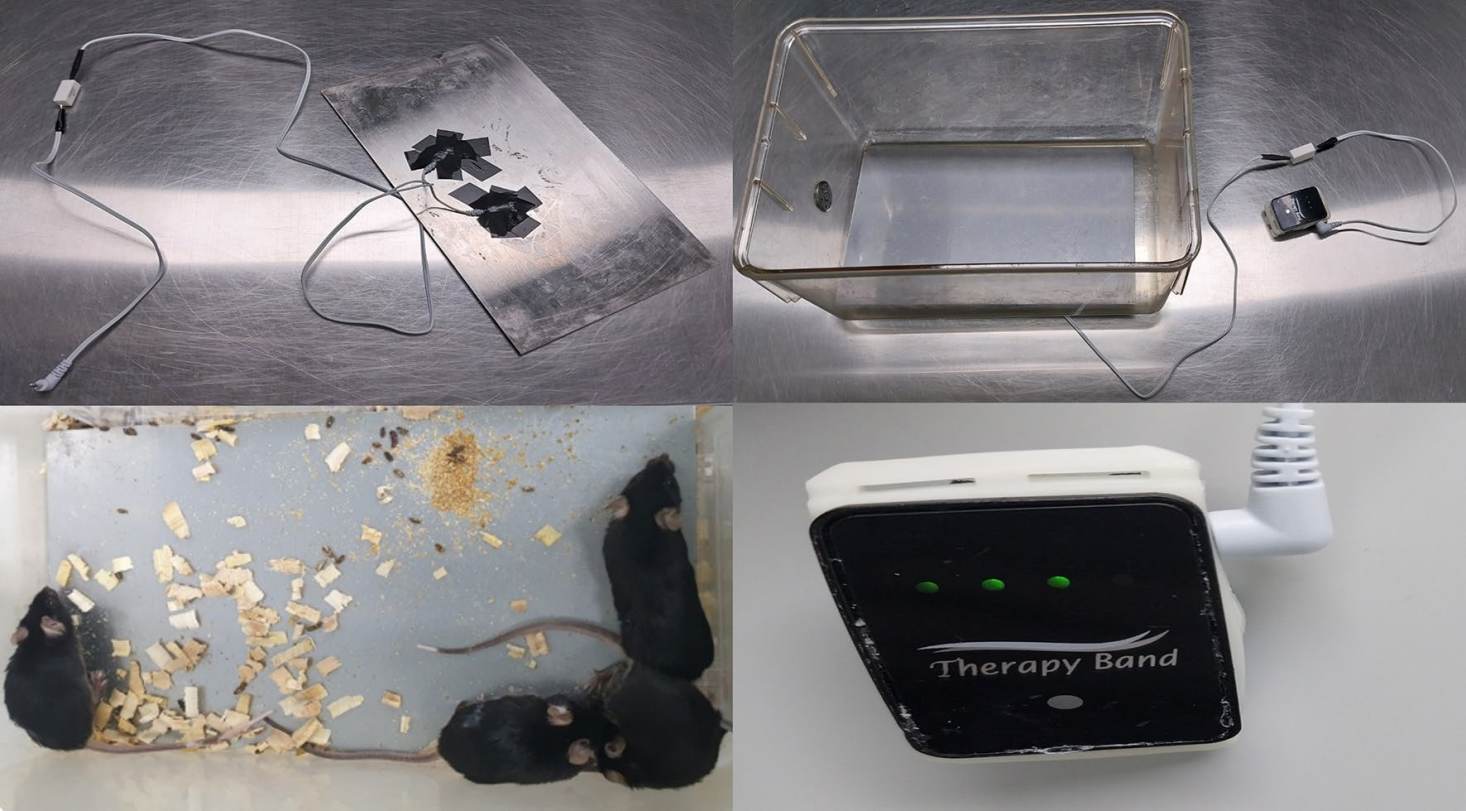
Microcurrent therapy. A connection wire from the microcurrent generator was used to deliver the current through a copper plate of the same size as the cage floor. This setup enabled the mice to receive the current through their feet touching the floor, allowing the current to reach the brain.

Microcurrent was applied for 12 h a day on weekdays according to the specific period set for each group to investigate its therapeutic effect (Figs. 4, 5C). A connection wire from the microcurrent generator (Natural Well Tech, Busan, Korea) was used to deliver the current through a copper plate of the same size as the cage floor. This setup enabled the mice to receive the current through their feet touching the floor, thereby allowing the current to reach the brain (Fig. 6). Microcurrent is usually applied during the active time of humans, which is daytime, thus microcurrent was applied during the mice’s active time, which is the nighttime cycle for nocturnal animals. There are various waveforms of microcurrent, but Kim et al.^47^ revealed that all waveforms had an effect, and the step form waveform showed significant effects on both clinical parameters, such as cognition and protein production related to Alzheimer’s disease in mice models. Therefore, we selected the microcurrent with the step form waveform (0, 1.5, 3, and 5 V) with wave superposition. The intensity of the microcurrent was set to 1 μ A (250 Ω), the voltage was set to 5 V, and the basic frequency was set to 7 Hz with an additional 44 KHZ frequency superimposition.

### Histopathologic analysis

All the histological parameters of the study were measured by an examiner (M.H.P.) who was unaware of the group allocation. Specimens were paraffin-embedded after at least one day of PFA fixation. The paraffin blocks were then sliced into five sections based on the location of the circle of Willis of cerebral arteries. H&E staining was performed to measure the vessel thickness and diameter for morphological examination. IHC was also conducted to analyze elastin, collagen, and SMCs using corresponding staining, such as Verhoeff Van Gieson, Masson’s trichrome, and alpha-smooth muscle actin (α-SMA), respectively. Furthermore, IHC of CD68, TNF-α, nuclear factor kappa-light-chain-enhancer of activated B cells (NF-κB), and MMP8 were conducted to examine inflammatory responses in the cerebral arteries. After mounting the tissue on the side, they were scanned by a Panoramic slide scanner to magnify H&E 200 × and IHC 400 × .

A scoring system was introduced to categorize the expression levels of pro-inflammatory mediators (CD68, TNF-α, NF-κB, and MMP-8) in the cerebral arterial wall. Grade 0 denoted the absence of marker expression in the cerebral artery, grade 1 indicated a marker expression below the halfway point of the arterial wall circumference, and grade 2 represented a marker expression above the halfway point, encompassing more than half of the arterial wall circumference.

### Measurement of diameter and thickness

**Figure 7:**
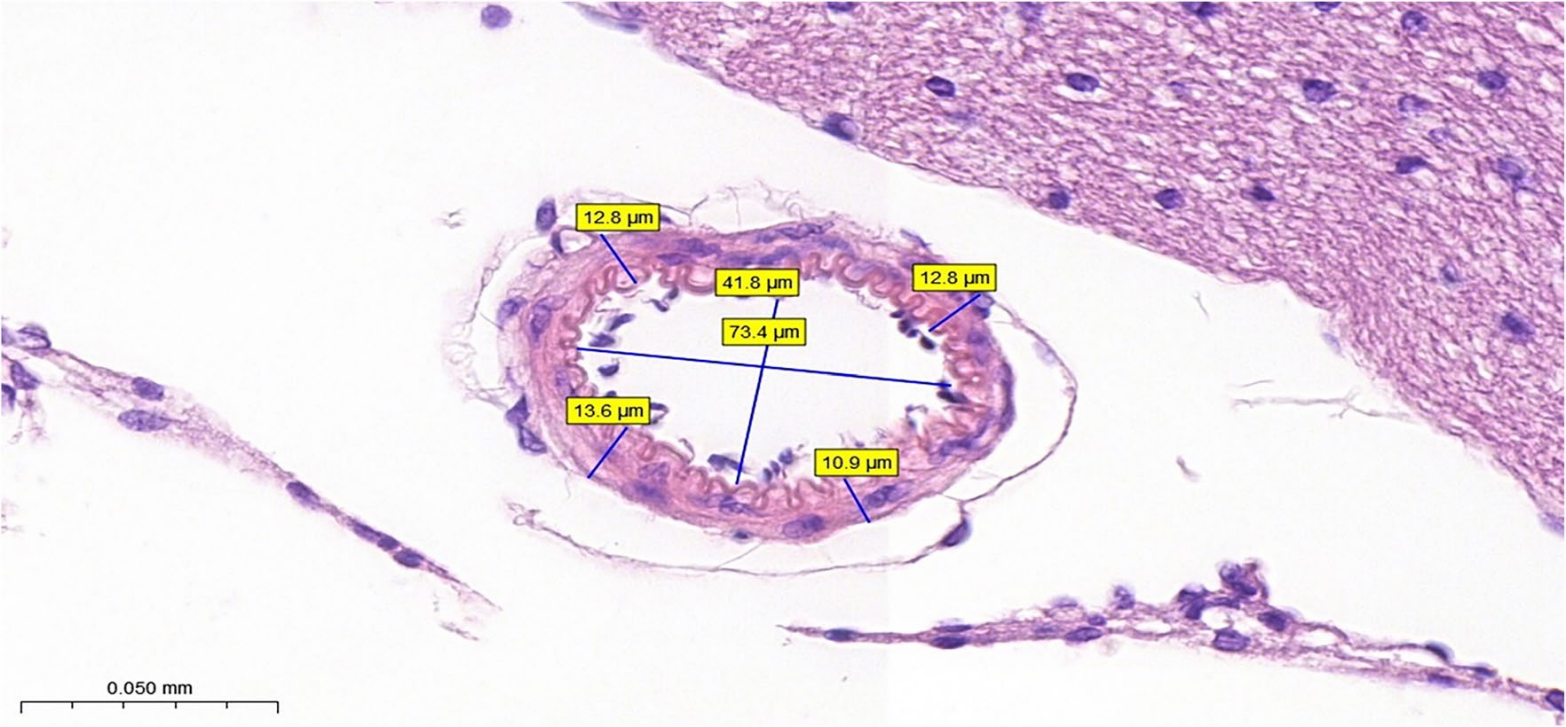
Measurements of cerebral artery lumen diameter and wall thickness. The length of the lumen diameter was calculated as the average value of the representative length of two horizontal points and two vertical points of the vessel, and the wall thickness was calculated as the average value of the representative thickness of each part of the vessel equally divided into four parts.

A ruler within the Panoramic Slide Scanner viewer program was used to measure the arterial diameter. The length of the cerebral arterial diameter was calculated by taking the average value between two horizontal points and two vertical points along the vessel. The cerebral arterial thickness was determined by dividing each vessel into four equal parts. The average value of the representative thickness in each part was calculated, providing an overall measurement of the vessel’s thickness (Fig. 7).

### Analysis of extracellular matrix components

The analysis was performed following the method presented in the previous study^1^. After removing the background using the Adobe Photoshop program, the extracellular matrix components of the cerebral arterial wall, such as SMCs, elastin, and collagen, were assessed by measuring the selected ROI within the image using ImageJ software. The area of the ROI was measured and then divided by the area of the entire cerebral artery to quantify the components. This calculation enabled the estimation of the percentage of each component present in the cerebral artery.

### Statistical analysis

Statistical analyses were conducted using the IBM Statistical Package for the Social Sciences program for Windows version 22.0 (IBM Corp., Armonk, New York, USA). For calculating sample size, we conducted pilot study. The primary end point is the cerebral arterial diameter. In pilot study, we used one mouse in each group, therefore four randomly selected field was evaluated in each group. The effect size was 0.49, and for such effect size, to achieve a power of at least 95% using the ANOVA with a significance level of 0.05, at least 76 fields were needed. Four fields can be obtained from one mouse. Therefore, 16 mouse were needed. Considering drop rate as 20%, we determined sample size as 20. One-way analysis of variance (ANOVA) was used to determine intra- and inter-group statistical differences. A post hoc Tukey test was additionally performed when one-way ANOVA demonstrated significant differences between the groups. The mean values were followed by 95% confidence intervals, and all the data were expressed as the means ± standard deviations. The statistically significant levels were pre-determined at *P*-values of < 0.05. The post-hoc power analysis was performed, and the power was calculated as > 0.95.

## Ethical statement

The authors are accountable for all aspects of the work in ensuring that questions related to the accuracy or integrity of any part of the work are appropriately investigated and resolved. The study was conducted according to the guidelines of the IACUC, and approved by the Catholic University of Daegu School of Medicine Animal Care and Use Committee.

Values are presented as mean ± standard deviation.

SMC: smooth muscle cell.

*p < 0.05, one-way ANOVA test among groups.

^†^*p* < 0.05, post hoc Tukey test between groups 1 and 2.

^‡^*p* < 0.05, post hoc Tukey test between groups 2 and 3.

^♦^*p* < 0.05, post hoc Tukey test between groups 2 and 4.

### Supplementary Information

Supplementary Information.

## Data Availability

All data generated or analyzed during this study are included in this published article.
